# Analysis of Outcomes Associated With Outpatient Management of Nonoperatively Treated Patients With Appendicitis

**DOI:** 10.1001/jamanetworkopen.2022.20039

**Published:** 2022-07-01

**Authors:** Writing Group for the CODA Collaborative, David A. Talan, Gregory J. Moran, Anusha Krishnadasan, Sarah E. Monsell, Brett A. Faine, Lisandra Uribe, Amy H. Kaji, Daniel A. DeUgarte, Wesley H. Self, Nathan I. Shapiro, Joseph Cuschieri, Jacob Glaser, Pauline K. Park, Thea P. Price, Nicole Siparsky, Sabrina E. Sanchez, David A. Machado-Aranda, Jesse Victory, Patricia Ayoung-Chee, William Chiang, Joshua Corsa, Heather L. Evans, Lisa Ferrigno, Luis Garcia, Quinton Hatch, Marc D. Horton, Jeffrey Johnson, Alan Jones, Lillian S. Kao, Anton Kelly, Daniel Kim, Matthew E. Kutcher, Mike K. Liang, Nima Maghami, Karen McGrane, Elizaveta Minko, Cassandra Mohr, Miriam Neufeld, Joe H. Patton, Colin Rog, Amy Rushing, Amber K. Sabbatini, Matthew Salzberg, Callie M. Thompson, Aleksandr Tichter, Jon Wisler, Bonnie Bizzell, Erin Fannon, Sarah O. Lawrence, Emily C. Voldal, Danielle C. Lavallee, Bryan A. Comstock, Patrick J. Heagerty, Giana H. Davidson, David R. Flum, Larry G. Kessler

**Affiliations:** 1 Department of Emergency Medicine, Ronald Reagan UCLA Medical Center, Los Angeles, California; 2 Department of Emergency Medicine, Olive View–UCLA Medical Center, Los Angeles, California; 3 Center for Biostatistics, University of Washington, Seattle; 4 College of Pharmacy, University of Iowa Hospitals and Clinics, Iowa City; 5 Department of Emergency Medicine, Harbor–UCLA Medical Center, West Carson, California; 6 Department of Pediatric General Surgery, Harbor–UCLA Medical Center, West Carson, California; 7 Department of Emergency Medicine, Vanderbilt University Medical Center, Nashville, Tennessee; 8 Department of Emergency Surgery, Beth Israel Deaconess Medical Center, Boston, Massachusetts; 9 Department of Surgery, Harborview Medical Center, UW Medicine, Seattle, Washington; 10 Department of Surgery, University of California, San Francisco; 11 Department of Surgery, Providence Regional Medical Center Everett, Everett, Washington; 12 Department of Surgery, Michigan Medicine, Ann Arbor; 13 Department of Surgery, Rush University Medical Center, Chicago, Illinois; 14 Department of Surgery, Boston University Medical Center, Boston, Massachusetts; 15 Department of Surgery, Bellevue Hospital Center, NYU School of Medicine, New York, New York; 16 Department of Surgery, Tisch Hospital, NYU Langone Medical Center, New York, New York; 17 Department of Surgery Morehouse School of Medicine, Atlanta, Georgia; 18 Department of Surgery, The Medical University of South Carolina, Charleston; 19 Department of Surgery, UCHealth University of Colorado Hospital, Denver; 20 Department of Surgery, University of Iowa Hospitals and Clinics, Iowa City; 21 Department of Surgery, Madigan Army Medical Center, Tacoma, Washington; 22 Department of Surgery, The Swedish Medical Center, Seattle, Washington; 23 Department of Surgery, Henry Ford Health System, Detroit, Michigan; 24 Department of Emergency Medicine, The University of Mississippi Medical Center, Jackson; 25 Department of Surgery, McGovern Medical School, The University of Texas Health Science Center at Houston; 26 Department of Surgery, Weill Cornell Medical Center, New York, New York; 27 Department of Surgery, University of Washington, Seattle; 28 Department of Surgery, The University of Mississippi Medical Center, Jackson; 29 Department of Surgery, Lyndon B. Johnson General Hospital, University of Texas, Houston; 30 Department of Surgery, University of Houston, HCA Healthcare, Kingwood, Kingwood, Texas; 31 Department of Surgery, Columbia University Medical Center, New York, New York; 32 Department of Surgery, The Ohio State University Wexner Medical Center, Columbus; 33 Department of Surgery, UH Cleveland Medical Center, Cleveland, Ohio; 34 Department of Emergency Medicine, University of Washington, Seattle; 35 Department of Emergency Medicine, UCHealth University of Colorado Hospital, Denver; 36 Department of Surgery, Vanderbilt University Medical Center, Nashville, Tennessee; 37 Department of Surgery, University of Utah, Salt Lake City; 38 Department of Emergency Medicine, Columbia University Medical Center, New York, New York; 39 Department of Emergency Medicine, Baylor College of Medicine, Houston, Texas; 40 BC Support Unit, BC Academic Health Science Network, Vancouver, British Columbia, Canada; 41 School of Public Health, University of Washington, Seattle

## Abstract

**Importance:**

In the Comparison of Outcomes of Antibiotic Drugs and Appendectomy (CODA) trial, which found antibiotics to be noninferior, approximately half of participants randomized to receive antibiotics had outpatient management with hospital discharge within 24 hours. If outpatient management is safe, it could increase convenience and decrease health care use and costs.

**Objective:**

To assess the use and safety of outpatient management of acute appendicitis.

**Design, Setting, and Participants:**

This cohort study, which is a secondary analysis of the CODA trial, included 776 adults with imaging-confirmed appendicitis who received antibiotics at 25 US hospitals from May 1, 2016, to February 28, 2020.

**Exposures:**

Participants randomized to antibiotics (intravenous then oral) could be discharged from the emergency department based on clinician judgment and prespecified criteria (hemodynamically stable, afebrile, oral intake tolerated, pain controlled, and follow-up confirmed). Outpatient management and hospitalization were defined as discharge within or after 24 hours, respectively.

**Main Outcomes and Measures:**

Outcomes compared among patients receiving outpatient vs inpatient care included serious adverse events (SAEs), appendectomies, health care encounters, satisfaction, missed workdays at 7 days, and EuroQol 5-dimension (EQ-5D) score at 30 days. In addition, appendectomy incidence among outpatients and inpatients, unadjusted and adjusted for illness severity, was compared.

**Results:**

Among 776 antibiotic-randomized participants, 42 (5.4%) underwent appendectomy within 24 hours and 8 (1.0%) did not receive their first antibiotic dose within 24 hours, leaving 726 (93.6%) comprising the study population (median age, 36 years; range, 18-86 years; 462 [63.6%] male; 437 [60.2%] White). Of these participants, 335 (46.1%; site range, 0-89.2%) were discharged within 24 hours, and 391 (53.9%) were discharged after 24 hours. Over 7 days, SAEs occurred in 0.9 (95% CI, 0.2-2.6) per 100 outpatients and 1.3 (95% CI, 0.4-2.9) per 100 inpatients; in the appendicolith subgroup, SAEs occurred in 2.3 (95% CI, 0.3-8.2) per 100 outpatients vs 2.8 (95% CI, 0.6-7.9) per 100 inpatients. During this period, appendectomy occurred in 9.9% (95% CI, 6.9%-13.7%) of outpatients and 14.1% (95% CI, 10.8%-18.0%) of inpatients; adjusted analysis demonstrated a similar difference in incidence (−4.0 percentage points; 95% CI, −8.7 to 0.6). At 30 days, appendectomies occurred in 12.6% (95% CI, 9.1%-16.7%) of outpatients and 19.0% (95% CI, 15.1%-23.4%) of inpatients. Outpatients missed fewer workdays (2.6 days; 95% CI, 2.3-2.9 days) than did inpatients (3.8 days; 95% CI, 3.4-4.3 days) and had similar frequency of return health care visits and high satisfaction and EQ-5D scores.

**Conclusions and Relevance:**

These findings support that outpatient antibiotic management is safe for selected adults with acute appendicitis, with no greater risk of complications or appendectomy than hospital care, and should be included in shared decision-making discussions of patient preferences for outcomes associated with nonoperative and operative care.

**Trial Registration:**

ClinicalTrials.gov Identifier: NCT02800785

## Key Points

**Question:**

Is outpatient management with hospital discharge within 24 hours safe among adults receiving antibiotic treatment for acute appendicitis?

**Findings:**

In this cohort study, which is a secondary analysis of the Comparison of Outcomes of Antibiotic Drugs and Appendectomy trial, that included 726 participants randomized to receive antibiotics, 46% who were judged to meet stability criteria and were discharged within 24 hours had less than 1 serious adverse event per 100 participants through 7 days. Along with subsequent appendectomies, serious adverse events occurred no more frequently in these patients than among hospitalized patients.

**Meaning:**

These findings support that outpatient antibiotic management is safe for selected adults with acute appendicitis and should be included in shared decision-making discussions of patient preferences for outcomes associated with nonoperative and operative care.

## Teaser

This cohort study assesses whether patients with acute appendicitis can be safely treated with antibiotics in the outpatient setting.

## Introduction

The Comparison of Outcomes of Antibiotic Drugs and Appendectomy (CODA) trial was the largest reported randomized clinical trial comparing antibiotic treatment and appendectomy for patients with imaging-confirmed appendicitis.^1^ The CODA trial found antibiotics to be noninferior to appendectomy for a 30-day general health measure, the EuroQol 5-dimension (EQ-5D) score. In addition, several secondary outcomes were compared, including serious adverse events (SAEs), health care encounters, and missed work; the subsequent appendectomy rate was reported among antibiotic-assigned participants. The CODA trial included patients with appendicolith, and SAE and appendectomy rates were greater in this subgroup.

One distinctive feature of the CODA trial was that an antibiotic-assigned participant who met stability criteria could be discharged from the emergency department (ED) with oral antibiotics after initial receipt of parenteral antibiotics. Outpatient antibiotic management has been described, to our knowledge, in only 1 small trial.^2^ Because nearly half of the participants randomized to receive antibiotics were discharged from the ED rather than admitted to hospital, the CODA trial allows an opportunity to better characterize this management approach.

In 2020, after publication of initial CODA trial results, the American College of Surgeons issued guidelines stating that high-quality evidence indicates that most patients can be treated with antibiotics rather than appendectomy.^3^ Outpatient management, if safe and effective, potentially affords greater patient convenience and reduced health care use and costs.^4^ Alternatively, if outpatient management is associated with clinical worsening related to oral antibiotic nonadherence and delayed surgical interventions, with more visits, complications, and appendectomies, then initial hospitalization may be the preferred strategy for antibiotic delivery and close observation. Therefore, we conducted a cohort study that analyzed participants randomized to receive antibiotics in the CODA trial to assess the use and safety of outpatient management across the spectrum of patients, illness presentations, and investigative sites.

## Methods

### Design

The CODA trial was a 25–US site, pragmatic, nonblinded, noninferiority, randomized clinical trial among patients 18 years or older with acute appendicitis to determine whether antibiotics were noninferior to appendectomy. Trial details have been previously published.^1^ The CODA trial protocol was approved by institutional review boards at all 25 participating sites. All participants provided written informed consent.

In this cohort study of participants randomized to receive antibiotics, we conducted the following secondary analyses: (1) description of outpatient management use by site; (2) comparison of characteristics, safety, and patient-related outcomes of participants receiving outpatient and hospital care; (3) comparison of outpatient- and inpatient-treated participant subsequent appendectomy rates, including adjustment for differences in measured baseline illness severity characteristics; and (4) description of overall appendectomy rates by proportion of antibiotic-assigned participants treated as outpatients at each site. This study is reported following the Strengthening the Reporting of Observational Studies in Epidemiology (STROBE) reporting guideline.

### Study Population

From May 1, 2016, to February 28, 2020, a total of 1552 adults with clinically suspected and imaging-confirmed localized appendicitis were randomized to antibiotic treatment or appendectomy (n = 776 each). Patients with diffuse peritonitis, severe sepsis or septic shock, an immunocompromising condition, pregnancy, inflammatory bowel disease, and imaging-identified large phlegmon or abscess, free air, or mass were excluded.^1^ Participants with imaging-identified appendicolith were analyzed as a prespecified subgroup. Participants who underwent appendectomy or did not receive their first antibiotic dose within 24 hours of ED registration were not candidates for ED discharge and were not included in this analysis.

### Interventions

Protocol-specified antibiotics consisted of a minimum of a once-daily intravenous regimen (eg, ertapenem or ceftriaxone and high-dose metronidazole) or scheduled doses with coverage for 24 hours followed by oral antibiotics to complete a 10-day total course. Clinical teams selected antibiotics based on guideline recommendations for intra-abdominal infections from the Surgical Infection Society and the Infectious Diseases Society of America.^5,6^ Discharge criteria, including from the ED, were specified in the study protocol as follows: (1) the patient was stable and had stable and near-normal vital signs; (2) the patient was afebrile; (3) the patient’s symptoms were controlled with oral analgesics; (4) the patient could tolerate oral fluids and medications; (5) the patient and clinician agreed that discharge was acceptable; and (6) a timely outpatient follow-up visit was confirmed. Interpretation of these criteria and the decision for outpatient management was at the discretion of the patient’s treating physicians. Participants could be cared for solely in the ED or subsequently in an observation unit or hospital based on physician discretion and availability of these care sites. Appendectomy was recommended for participants who developed diffuse peritonitis and/or severe sepsis or septic shock at any time or who experienced worsening symptoms after 48 hours of antibiotic use. However, these criteria were not required for appendectomy to be performed, and the treating clinician and participant ultimately decided on an appendectomy.

### Definitions

We defined outpatient management as occurring if the patient was discharged within 24 hours of ED registration (typically from the ED but occasionally from other care areas) and defined hospitalization as a stay longer than 24 hours. The 24-hour threshold was based on the time by which participants had imaging performed, diagnosis confirmed, and antibiotic doses administered such that they could be considered for outpatient care, prior outpatient management study definitions,^7^ and Centers for Medicare & Medicaid Services (CMS) criteria. The CMS stipulates that outpatient observation services generally do not exceed 24 hours, and the 2-midnight rule qualifies more than 24 hours of hospital care as inpatient admission.^8,9^ In addition to cost implications, care beyond 24 hours would require an overnight stay that would be inconsistent with outpatient care. Because of variation in availability of observation and hospital beds and physician discretion, some antibiotic-treated participants had ED discharge after 24 hours. We previously reported that 365 of 776 antibiotic-randomized participants (47.0%) had ED discharge at any time after ED registration^1^; 30 participants (3.8%) had ED discharge after 24 hours. For this analysis and the reasons mentioned in this section, participants with ED discharge after 24 hours were considered to have been hospitalized.

### Outcome Measures

The primary outcome was safety as determined by SAE incidence. An SAE was defined as death, a life-threatening event, significant treatment-related disability or incapacity, or non–appendicitis-related hospitalization.^1^ The incidence of SAEs was primarily examined among outpatient-treated participants but was also compared with that of hospitalized participants, along with other outcomes. Other outcomes included the following: (1) appendectomies, (2) later hospitalization and ED and urgent care visits, (3) missed workdays, (4) National Surgical Quality Improvement Program events,^10^ (5) patient-reported treatment dissatisfaction, and (6) EQ-5D score. The primary outcome interval was 0 to 7 days based on the assumption that antibiotic failure would affect early clinical response. We also evaluated outcomes through 30 days, as well as appendectomies through 2 years, among subgroups with ED discharge between 0 and 12 hours and greater than 12 and 24 hours, and among those with an appendicolith. The EQ-5D scores were only assessed at 30 days. The instrumental support score was determined by Patient-Reported Outcomes Measurement Information System (PROMIS) for the outcomes of having someone to take the patient to a physician, run errands, and help with daily chores and if confined to bed.^11^

### Statistical Analysis

We described participant baseline characteristics and outcomes, stratified by time from ED registration to discharge within 24 hours and a stay of longer than 24 hours, with subgroups for discharge between 0 and 12 hours and between more than 12 and up to 24 hours, using frequency (SAEs per 100 participants and for other outcomes as percentage of participants with 95% CIs) for categorical data and means (SDs) for continuous measures. We calculated Kaplan-Meier–based cumulative incidence of appendectomy through 30 days and through 2 years stratified by discharge at 24 hours or less and discharge at longer than 24 hours.

We evaluated outcomes unadjusted for differences in measured baseline characteristics of participants treated as outpatients and inpatients. We knew that participants discharged within 24 hours should have met specific criteria, but our data did not capture their presence or absence or specific findings, such as vital signs. Therefore, we conducted an exploratory analysis of occurrence of appendectomies adjusting for measured baseline characteristics that may have differed across the 2 groups and that were potentially associated with illness severity that may have affected the clinician’s decision to discharge or hospitalize. We recognize that our adjustment did not address unmeasured differences, such as change in clinical symptoms, which were integral to the protocol discharge criteria, and between and within investigative sites.

We used multiple imputations with chained equations to address missing data in baseline characteristics and appendectomy status at 7 days for comparison of risk differences of outpatients and inpatients. Adjustment variables were selected based on investigator consensus and included the following: participant age, Charlson Comorbidity Index, history of fever (yes/no), history of nausea or vomiting (yes/no), Alvarado score, maximum pain score in the ED (10-point scale, with 1 indicating no pain and 10 indicating the most severe pain), initial white blood cell count, appendicolith on imaging (yes/no), and appendiceal diameter on imaging. These characteristics and others were included in the imputation process (eAppendix in Supplement 1). Model coefficients were marginalized and pooled across the 10 imputation sets.^12^ The number of SAEs was insufficient to perform a covariate-adjusted analysis. Unadjusted and adjusted risk differences are shown with corresponding 95% CIs, estimated using a generalized linear model with the binomial link function. We also conducted an ecological analysis to explore the association between outpatient management and overall appendectomy rate by site. All analyses were performed in R statistical software, version 4.0.3 (R Foundation for Statistical Computing).

## Results

Among 776 antibiotic-randomized participants, 42 (5.4%) underwent appendectomy within 24 hours (characteristics described in the eTable in Supplement 1) and 8 (1.0%) did not receive their first antibiotic dose within 24 hours, leaving 726 (93.6%) in the study population (median age, 36 years; range, 18-86 years; 462 [63.6%] male; 68 [9.4%] African American or Black, 12 [1.7%] American Indian or Alaska Native, 35 [4.8%] Asian, 4 [0.5%] Native Hawaiian or other Pacific Islander, 437 [60.2%] White, 25 [3.4%] of multiple races, and 138 [19.0%] of other race [race selected by the participants if they did not identify themselves with any of the listed races]) (eFigure 1 in Supplement 1). Of these 726 antibiotic-treated participants, 335 (46.1%) were discharged within 24 hours (163 at 0-12 hours and 172 at >12-24 hours), and 391 (53.9%) were discharged after 24 hours.

**Figure 1.**
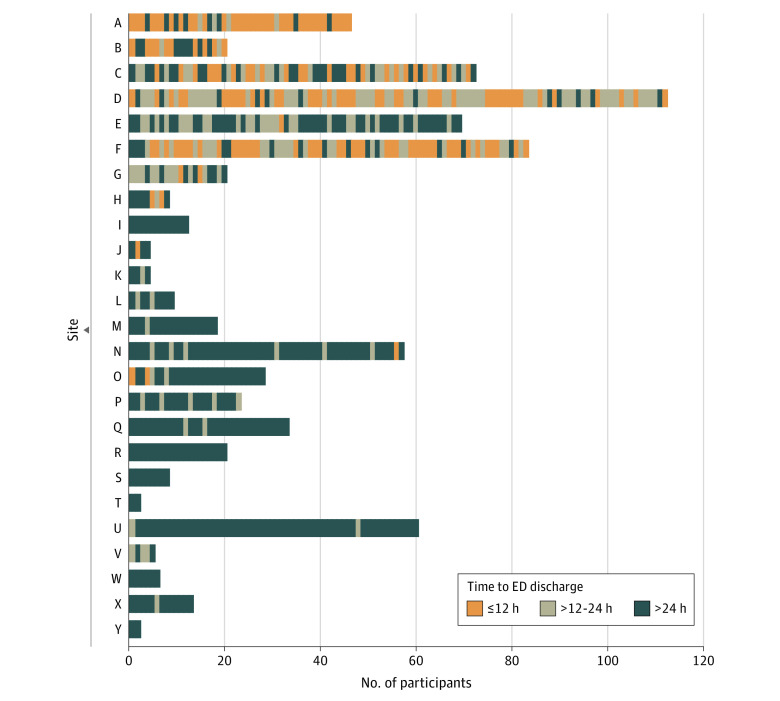
Time to Emergency Department (ED) Discharge of Sequentially Enrolled Participants Randomized to Receive Antibiotics at 25 US Comparison of Outcomes of Antibiotic Drugs and Appendectomy Trial Sites

Time to discharge of sequentially enrolled antibiotic-randomized participants at 25 sites is shown in Figure 1. Characteristics of participants who were discharged at 0 to 24 hours (and the 0- to 12-hour and >12- to 24-hour subgroups) and after 24 hours are given in Table 1. Participants receiving outpatient management less often identified as African American or Black and more often as other nonlisted races; otherwise, the groups were similar for other characteristics, including for age, pain, Alvarado scores, white blood cell count, frequency of imaging-identified appendicolith, and appendiceal diameter.

**Table 1.** Characteristics of Comparison of Outcomes of Antibiotic Drugs and Appendectomy Trial Participants Randomized to Receive Antibiotics by Time to Hospital Discharge

Characteristic	**Participants, No. (%) [95% CI]**			
	0-24 h (n = 335)	>24 h (n = 391)	0-12 h (n = 163)	>12-24 h (n = 172)
Age, y				
<30	100 (29.9) [25.0-35.1]	140 (35.8) [31.1-40.8]	54 (33.1) [26.0-40.9]	46 (26.7) [20.3-34.0]
30-39	89 (26.6) [21.9-31.6]	119 (30.4) [25.9-35.3]	45 (27.6) [20.9-35.1]	44 (25.6) [19.2-32.8]
40-49	71 (21.2) [16.9-26.0]	67 (17.1) [13.5-21.2]	33 (20.3) [14.4-27.2]	38 (22.1) [16.1-29.0]
50-59	50 (14.9) [11.3-19.2]	37 (9.5) [6.8-12.8]	22 (13.5) [8.7-19.7]	28 (16.3) [11.1-22.7]
60-69	19 (5.7) [3.7-8.7]	22 (5.6) [3.6-8.4]	8 (4.9) [2.1-9.4]	11 (6.4) [3.2-11.1]
70-79	6 (1.8) [0.6-3.9]	5 (1.3) [0.4-3.0]	1 (0.6) [0.0-3.4]	5 (2.9) [1.0-6.7]
≥80	0	1 (0.3) [0.0-1.4]	0	0
Sex				
Male	213 (63.6) [58.2-68.7]	249 (63.7) [58.7-68.5]	117 (71.8) [64.2-78.5]	96 (55.8) [48.1-63.4]
Female	122 (36.4) [31.3-41.8]	142 (36.3) [31.5-41.3]	46 (28.2) [21.5-35.8]	76 (44.2) [36.6-51.9]
Race				
African American or Black	13 (3.9) [2.1-6.5]	55 (14.1) [10.8-17.9]	6 (3.7) [1.4-7.8]	7 (4.1) [1.7-8.2]
American Indian or Alaska Native	0	12 (3.1) [1.6-5.3]	0	0
Asian	22 (6.6) [4.2-9.8]	13 (3.3) [1.8-5.6]	13 (8.0) [4.3-13.3]	9 (5.2,2.4-9.7]
Native Hawaiian or other Pacific Islander	4 (1.2) [0.3-3.0]	0	1 (0.6) [0.0-3.4]	3 (1.7) [0.4-5.0]
White	195 (58.2) [52.7-63.6]	242 (61.9) [56.9-66.7]	96 (58.9) [50.9-66.5]	99 (57.6) [49.8-65.1]
Other race^a^	88 (26.2) [21.6-31.3]	50 (12.8) [9.6-16.5]	38 (23.3) [17.1-30.6]	50 (29.1) [22.4-36.5]
Multiple races	10 (3.0) [1.4-5.4]	15 (3.8) [2.2-6.3]	8 (4.9) [2.1-9.4]	2 (1.2) [0.1-4.1]
Hispanic				
No	174 (51.9) [46.4-57.4]	212 (54.2) [49.1-59.2]	90 (55.2) [47.2-63.0]	84 (48.8) [41.2-56.6]
Yes	161 (48.1) [42.6-53.6]	179 (45.8) [40.8-50.9]	73 (44.8) [37.0-52.8]	88 (51.2) [43.4-58.9]
Primary language				
English	196 (58.5) [53.0-63.8]	239 (61.1) [56.1-66.0]	97 (59.5) [51.6-67.1]	99 (57.6) [49.8-65.1]
Spanish	120 (35.8) [30.7-41.2]	134 (34.3) [29.6-39.2]	54 (33.1) [26.0-40.9]	66 (38.4) [31.1-46.1]
Other	19 (5.7) [3.5-8.7]	18 (4.6) [2.8-7.2]	12 (7.4) [3.9-12.5]	7 (4.1) [1.7-8.2]
Health insurance				
Commercial	144 (43.0) [37.6-48.5]	163 (41.7) [36.8-46.8]	75 (46.0) [38.2-54.0]	69 (40.1) [32.7-47.9]
Medicare or Tricare	46 (13.7) [10.3-17.9]	38 (9.7) [7.0-13.1]	21 (12.9) [8.2-19.0]	25 (14.5) [9.6-20.7]
Medicaid or state	56 (16.7) [12.9-21.2]	67 (17.1) [13.5-21.2]	23 (14.1) [9.2-20.4]	33 (19.2) [13.6-25.9]
Other or none	87 (26.0) [21.4-31.0]	110 (28.1) [23.7-32.9]	43 (26.4) [19.8-33.8]	44 (25.6) [19.2-32.8]
Health literacy help^b^				
Never or rarely	264 (82.2) [77.6-86.3]	305 (80.3) [75.9-84.2]	126 (81.8) [74.8-87.6]	138 (82.6) [76.0-88.1]
Sometimes or more	57 (17.8) [13.7-22.4]	75 (19.7) [15.9-24.1]	28 (18.2) [12.4-25.2]	29 (17.4) [12.0-24.0]
Below federal poverty level or receiving Medicaid^b^				
No	140 (54.1) [47.8-60.2]	155 (53.6) [47.7-59.5]	70 (53.4) [44.5-62.2]	70 (54.7) [45.7-63.5]
Yes	119 (46.0) [39.8-52.2]	134 (46.4) [40.5-52.3]	61 (46.6) [37.8-55.5]	58 (45.3) [36.5-54.4]
Dependents^b^				
No	115 (35.2) [30.0-40.6]	148 (39.0) [34.0-44.0]	52 (32.7) [25.5-40.6]	63 (37.5) [30.2-45.3]
Yes	212 (64.8) [59.4-70.0]	232 (61.1) [56.0-66.0]	107 (67.3) [59.4-74.5]	105 (62.5) [54.7-69.8]
BMI^b^				
<25	55 (29.4) [23.0-36.5]	115 (31.2) [26.5-36.2]	23 (28.8) [19.2-40.0]	32 (29.9) [21.4-39.5]
25-35	104 (56.6) [48.2-62.9]	195 (52.9) [47.6-58.0]	43 (53.8) [42.2-65.0]	61 (57.1) [47.1-66.5]
>35	28 (15.0) [10.2-20.9]	59 (16.0) [12.4-20.1]	14 (17.5) [9.9-27.6]	14 (13.1) [7.3-21.0]
Symptom duration, d				
<1	85 (25.4) [20.8-30.4]	99 (25.3) [21.1-29.9]	49 (30.1) [23.1-37.7]	36 (20.9) [15.1-27.8]
1-<2	116 (34.6) [29.5-40.0]	160 (40.9) [36.0-46.0]	52 (31.9) [24.8-39.7]	64 (37.2) [30.0-44.9]
≥2	134 (40.0) [34.7-45.5]	132 (33.8) [29.1-38.7]	62 (38.0) [30.6-46.0]	72 (41.9) [34.4-49.6]
Fever				
No	253 (75.5) [70.6-80.0]	292 (74.7) [70.1-78.9]	118 (72.4) [64.9-79.1]	135 (78.5) [71.6-84.4]
Yes	82 (24.5) [20.0-29.5]	99 (25.3) [21.1-29.9]	45 (27.6) [20.9-35.1]	37 (21.5) [15.6-28.4]
Nausea, vomiting, or anorexia				
No	62 (18.5) [14.5-23.1]	63 (16.1) [12.6-20.1]	30 (18.4) [12.8-25.2]	32 (18.6) [13.1-25.2]
Yes	273 (81.5) [76.9-85.5]	327 (83.6) [79.6-87.2]	133 (81.6) [74.8-87.2]	140 (81.4) [74.8-86.9]
CT only				
No	60 (17.9) [14.0-22.4]	83 (21.2) [17.3-25.6]	35 (21.5) [15.4-28.6]	25 (14.5) [9.6-20.7]
Yes	275 (82.1) [77.6-86.1]	308 (78.8) [74.4-82.7]	128 (78.5) [71.4-84.6]	147 (85.5) [79.3-90.4]
Ultrasonography only				
No	318 (94.9) [92.0-97.0]	385 (98.5) [96.7-99.4]	148 (90.8) [85.3-94.8]	170 (98.8) [95.9-99.9]
Yes	17 (5.1) [3.0-8.0]	6 (1.5) [0.6-3.3]	15 (9.2) [5.2-14.7]	2 (1.2) [0.1-4.1]
Appendicolith				
No	249 (74.3) [69.3-78.9]	283 (72.4) [67.7-76.8]	115 (70.6) [62.9-77.4]	134 (77.9) [71.0-83.9]
Yes	86 (25.7) [21.1-30.7]	108 (27.6) [23.3-32.3]	48 (29.5) [22.6-37.1]	38 (22.1) [16.1-29.0]
Perforation, abscess, or phlegmon^b^				
No or not mentioned	271 (85.0) [80.6-88.7]	332 (86.9) [83.1-90.1]	128 (87.1) [80.6-92.0]	143 (84.1) [77.7-89.3]
Yes	46 (14.4) [10.8-18.8]	50 (13.1) [9.9-16.9]	19 (12.9) [8.0-19.5]	27 (15.9) [10.7-22.3]
Instrumental support score, mean (95% CI)^b^^,^^c^	15 (15-16)	15 (15-16)	15 (15-16)	15 (15-16)
Charlson Index, mean (95% CI)^b^	0.3 (0.2-0.3)	0.2 (0.2-0.3)	0.2 (0.1-0.3)	0.3 (0.2-0.4)
Alvarado score, mean (95% CI)^b^	6.6 (6.5-6.8)	6.6 (6.4-6.7)	6.6 (6.3-6.8)	6.7 (6.5-7.0)
Pain score in the previous 7 d, mean (95% CI)	5.4 (5.1-5.7)	5.3 (5.0-5.6)	5.1 (4.7-5.6)	5.6 (5.2-6.1)
White blood cell count, mean (95% CI), /μL^a^	12 700 (12 300-13 100)	13 000 (12 600-13 400)	12 700 (12 100-13 400)	12 700 (12 100-13 200)
Appendiceal diameter on radiologic imaging, mean (95% CI), mm^b^	11 (11-12)	12 (11-12)	11 (11-12)	11 (11-12)

Abbreviations: BMI, body mass index (calculated as weight in kilograms divided by height in meters squared); CT, computed tomography.

SI conversion factor: To convert white blood cell count to ×10^9^/L, multiply by 0.001.

^a^ Other race was selected by the participants if they did not identify themselves with any of the listed races.

^b^ Participants with missing data were excluded from the denominator. Missing data occurred for participants as follows: health literacy help, 14 discharged within 24 hours and 11 discharged after 24 hours; below federal poverty level, 76 discharged within 24 hours and 102 discharged after 24 hours; dependents, 8 discharged within 24 hours and 11 discharged after 24 hours; BMI, 148 discharged within 24 hours and 22 discharged after 24 hours; perforation, abscess, or phlegmon, 18 discharged within 24 hours and 9 discharged after 24 hours; instrumental support, 13 discharged within 24 hours and 8 discharged after 24 hours; Charlson Comorbidity Index, 3 discharged within 24 hours; Alvarado score, 13 discharged within 24 hours and 23 discharged after 24 hours; pain score, 9 discharged within 24 hours and 11 discharged after 24 hours; white blood cell count, 3 discharged within 24 hours; and appendiceal diameter, 30 discharged within 24 hours and 69 discharged after 24 hours.

^c^ Instrumental support score was determined by the Patient-Reported Outcomes Measurement Information System (PROMIS) patient-related outcome of having someone to take the patient to the physician, run errands, and help with daily chores and if confined to bed.^11^

Outcomes by time of discharge are given in Table 2. Participants with discharge within 24 hours had 0.9 SAEs (95% CI, 0.2-2.6) per 100 participants during 7 days. Among those discharged after 24 hours, 7-day SAE incidence was 1.3 (95% CI, 0.4-2.9) per 100 participants. Serious adverse events occurred in 0.61 (95% CI, 0.02-3.4) per 100 discharged within 12 hours. In the appendicolith subgroup, SAEs occurred in 2.3 (95% CI, 0.3-8.2) per 100 participants discharged within 24 hours vs 2.8 (95% CI, 0.6-7.9) per 100 participants discharged after 24 hours during 7 days. All SAEs but 1 were attributable to subsequent hospitalization unrelated to appendicitis. Within 30 days, SAEs occurred in 1.8 (95% CI, 0.7-3.9) per 100 outpatients and 3.1 (95% CI, 1.6-5.4) per 100 inpatients, and no deaths occurred.

**Table 2.** Outcomes of Comparison of Outcomes of Antibiotic Drugs and Appendectomy Trial Participants Randomized to Receive Antibiotics by Time to Hospital Discharge

Outcome	Participants, No./total (%) [95% CI]			
	≤24 h	>24 h	≤12 h	12-24 h
SAEs^a^				
By 7 d	3/335 (0.9) [0.2-2.6]	5/391 (1.3) [0.4-2.9]	1/163 (0.61) [0.02-3.4]	2/172 (1.2) [0.1-4.2]
Subsequent hospitalization by 7 d	3/335 (0.9) [0.2-2.6]	4/391 (1.0) [0.3-2.6]	1/163 (0.61) [0.02-3.4]	2/172 (1.2) [0.14-4.2]
At least 1 SAE by 7 d	3/335 (0.9) [0.2-2.6]	5/391 (1.3) [0.4-3.0]	1/163 (0.6) [0.0-3.4]	2/172 (1.2) [0.1-4.1]
By 30 d	6/335 (1.8) [0.7-3.9]	14/391 (3.6) [2.0-6.0]	4/163 (2.5) [0.7-6.3]	2/172 (1.2) [0.1-4.2]
Subsequent hospitalization by 30 d	6/335 (1.8) [0.7-3.9]	12/391 (3.1) [1.6-5.4]	4/163 (2.5) [0.7-6.3]	2/172 (1.2) [0.1-4.2]
At least 1 SAE by 30 d	5/335 (1.5) [0.5-3.4]	12/391 (3.1) [1.6-5.3]	3/163 (1.8) [0.4-5.3]	2/172 (1.2) [0.1-4.1]
Appendectomy				
By 7 d	33/332 (9.9) [6.9-13.7]	55/389 (14.1) [10.8-18.0]	20/162 (12.3) [7.7-18.4]	13/170 (7.6) [4.1-12.7]
By 30 d	40/318 (12.6) [9.1-16.7]	70/368 (19.0) [15.1-23.4]	23/155 (14.8) [9.6-21.4]	17/163 (10.4) [6.2-16.2]
NSQIP events				
By 7 d	6/333 (1.8) [0.66-3.9]	22/391 (5.6) [3.5-8.5]	3/162 (1.9) [0.38-5.4]	3/171 (1.8) [0.36-5.1]
Site-related infectious complications^b^	4/335 (1.2) [0.3-3.0]	10/391 (2.6) [1.2-4.7]	3/163 (1.8) [0.4-5.3]	1/172 (0.6) [0.0-3.2]
Patient-reported time in health care after index				
Any overnight hospital stay after index care by 7 d	30/320 (9.4) [6.4-13.1]	20/349 (5.7) [3.5-8.7]	18/157 (11.5) [6.9-17.5]	12/163 (7.4) [3.9-12.5]
Any ED or UC visit after index care by 7 d	12/319 (3.8) [2.0-6.5]	4/350 (1.1) [0.31-2.9]	9/156 (5.8) [2.7-10.7]	3/163 (1.8) [0.4-5.3]
Days missed work through 7 d, mean (95% CI)^c^	2.6 (2.3-2.9)	3.8 (3.4-4.3)	2.4 (2.0-2.8)	2.8 (2.3-3.2)
Treatment dissatisfaction by 7 d	22/300 (7.3) [4.7-10.9]	32/331 (9.7) [6.7-13.4]	11/146 (7.5) [3.8-13.1]	11/154 (7.1) [0.4-12.4]
EQ-5D score at 30 d, mean (SD)^d^	0.93 (0.92-0.94)	0.92 (0.90-0.93)	0.93 (0.91-0.95)	0.93 (0.91-0.95)

Abbreviations: ED, emergency department; EQ-5D, EuroQol 5-dimension; NSQIP, National Surgical Quality Improvement Program; SAE, serious adverse event; UC, urgent care.

^a^ Two of 3 SAEs in the 24-hour or less group and 3 of 5 SAEs in the greater than 24-hour group occurred in patients who had an appendicolith on imaging.

^b^ Site-related infectious complications were defined as incisional infections or organ-space infections (abscesses) that had occurred at 30 days.

^c^ Numbers for missed work are 246 for time to discharge of 24 hours or less, 290 for time to discharge of greater than 24 hours, 125 for time to discharge of 12 hours or less, and 121 for time to discharge of 12 to 24 hours.

^d^ Numbers for EQ-5D are 306 for time to discharge of 24 hours or less, 337 for time to discharge of greater than 24 hours, 148 for time to discharge of 12 hours or less, and 158 for time to discharge of 12 to 24 hours.

**Figure 2.**
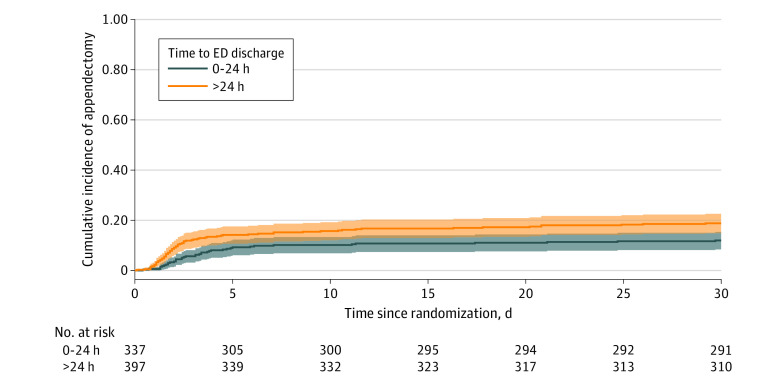
Cumulative Incidence of Appendectomy Through 30 Days in the Antibiotic Group by Emergency Department (ED) Discharge Within 24 Hours or Later

Appendectomies during 30 days for outpatient- and inpatient-treated participants are detailed in Figure 2 (and through 2 years in eFigure 2 in Supplement 1). At 7 days, 9.9% (95% CI, 6.9%-13.7%) of outpatients underwent an appendectomy compared with 14.1% (95% CI, 10.8%-18.0%) of inpatients. In the appendicolith subgroup, the 7-day appendectomy rate was 19.0% (95% CI, 11.3%-29.1%) among those discharged within 24 hours vs 23.4% (95% CI, 15.7%-32.5%) among those discharged after 24 hours. The unadjusted risk difference (with missing data imputed) for 7-day appendectomy rates for outpatients and inpatients was −4.6 percentage points (95% CI, −9.6 to 0.3), and the adjusted risk difference was −4.0 percentage points (95% CI, −8.7 to 0.6). At 30 days, appendectomy rates were 12.6% (95% CI, 9.1%-16.7%) for outpatients and 19.0% (95%, CI, 15.1%-23.4%) for inpatients.

**Figure 3.**
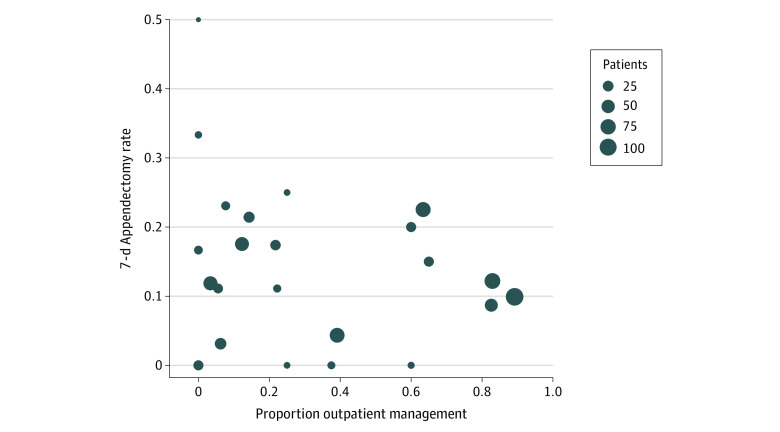
Overall Appendectomy Rate Through 7 Days and Proportion of Participants Randomized to Receive Antibiotics Who Were Discharged From the Emergency Department Within 24 Hours by Investigative Site

The proportion of participants discharged within 24 hours at each of the 25 investigative sites varied from 0.0% to 89.2% (median, 21.7%; IQR, 3.4%-57.9%). Figure 3 shows the association of appendectomy rate among all antibiotic-treated participants (inpatients and outpatients) during 7 days with the proportion receiving outpatient management at each site with more than 10 participants randomized to receive antibiotics.

Patient-related and other outcomes were also evaluated at 7 days (Table 2). Patients in both the outpatient and inpatient settings had low rates of treatment dissatisfaction. National Surgical Quality Improvement Program events occurred in 1.8 (95% CI, 0.8-3.9) per 100 outpatients and 5.6 (95% CI, 3.5-8.5) per 100 hospitalized participants. Later unplanned outpatient (ED or urgent care) visits occurred in 3.8% (95% CI, 2.0%-6.5%) of those with outpatient management vs 1.1% (95% CI, 0.3%-2.9%) who were hospitalized; hospitalization after the index visit care occurred in 9.4% (95% CI, 6.4%-13.1%) of outpatients vs 5.7% (95% CI, 3.5%-8.7%) of those who were hospitalized. Compared with those who were hospitalized, outpatients missed fewer workdays (2.6 days; 95% CI, 2.3-2.9 days) vs (3.8 days; 95% CI, 3.4-4.3 days). The EQ-5D-scores at 30 days were similarly high.

## Discussion

The CODA trial is the largest comparative study of antibiotics vs appendectomy for treatment of appendicitis to date, and it also provides the largest body of data on the experience of patients treated with antibiotics in the outpatient setting. In this trial, outpatient management among selected adult patients who met stability criteria was associated with rare SAEs (<1 event per 100 participants through 7 days), which occurred no more frequently than in hospitalized patients. Outpatient management appeared to be safe across a wide range of participants, including among those with an appendicolith and those judged able to be discharged within 12 hours of ED arrival.

Use of outpatient management varied greatly among investigative sites, from never to nearly 90% of nonoperatively treated participants. Those treated with antibiotics as outpatients did not have a greater frequency of appendectomy during 7 days than those hospitalized (9.9% vs 14.1%; during 30 days, 12.6% vs 19.0%). The difference in 7-day appendectomy incidence was similar when the analysis was adjusted for illness severity factors (between-group difference, −4.0 percentage points; 95% CI, −8.7 to 0.6). Trial sites that used outpatient management for most antibiotic-treated participants were not observed to have greater overall appendectomy rates than sites that used it less frequently. Less than 5% of outpatient-treated participants returned for unplanned outpatient care, and more than 90% avoided hospitalization through 7 days. Patient-related outcomes (7-day satisfaction rates and 30-day EQ-5D scores) were similarly high, and outpatients missed less work than inpatients.

Almost all past studies^13^ that compared antibiotics and appendectomy required hospitalization for patients treated nonoperatively. In 2017, Talan et al^2^ reported on a pilot randomized clinical trial of nonoperative vs operative treatment in which antibiotic-assigned adult participants could be discharged from the ED based on stability criteria similar to those of the CODA trial and after a minimum of 6 hours of observation. Participants had a next-day follow-up visit. In that trial, 14 of 15 antibiotic-treated participants (93.3%) received outpatient management; none required appendectomy during the next 30 days. The CODA trial neither specified a minimum observation period nor required next-day follow-up. However, participants usually had been treated for many hours before their diagnosis was established and antibiotics were given; on discharge, participants were closely followed up. In 2021, Sippola et al^14^ reported a trial of adults randomized to receive oral moxifloxacin for 7 days or intravenous ertapenem for 2 days and then oral levofloxacin and metronidazole for 5 days; 70.2% of the all-oral group and 73.8% of the intravenous-to-oral group did not undergo appendectomy at 1 year, although all-oral treatment was not shown to be noninferior. Participants were evaluated in the hospital twice daily, with the minimal observation of 20 to 24 hours before discharge. To our knowledge, no studies of outpatient management in children with appendicitis have been published.

In the current study, the safety of outpatient management compared with hospitalization may be related to differences in antibiotic delivery and adherence and timing of surgical care being insufficiently important to often result in SAEs. Patients discharged within 24 hours may have been at lower risk for complications because they were selected based on physician judgment and stability criteria. Hospitalization itself may have been associated with greater opportunity for interventions leading to SAEs, some of which may have been avoided with outpatient care.^15^

Compared with routine hospitalization, to the extent that outpatient management can be done among qualifying patients, our findings support that such management would not result in greater risk of SAEs or appendectomy and hence could be more convenient and less costly. Although outpatient surgery is possible, almost all patients undergoing appendectomy are currently hospitalized.^1,16^ Compared with inpatient care, outpatient antibiotic management was associated with 1 day less of missed work for participants. Furthermore, outpatients uncommonly had to return for unscheduled care visits or require hospitalization during the following 7 days, with these visits occurring no more frequently than among those who were initially admitted for hospital care. A US health sector perspective cost-effectiveness study^4^ found that antibiotic treatment with ED discharge was associated with less cost than hospital admission, amounting to more than $1000 per case and varying with length of stay. Differences in patient charges would be expected to be substantially greater, particularly if comparing those associated with outpatient antibiotic treatment and inpatient appendectomy.

Broader application of outpatient nonoperative management is suggested by similarity of characteristics between participants managed in the outpatient and inpatients settings and associated unadjusted and adjusted outcomes. In addition, overall appendectomy rates were not higher among high-enrolling CODA sites treating more than 75% of antibiotic-treated participants as outpatients compared with sites where this occurred less frequently. The site that most frequently practiced outpatient management (99 of 111 participants [89.2%]) was both the highest-enrolling CODA site and the location for the pilot study of this innovation; this finding could reflect higher use associated with greater physician experience and comfort with this approach.^2^ We found differences in the rate of outpatient management by racial identity that appeared site related; broader uptake of this care could help eliminate these disparities. Future studies should evaluate the extent to which outpatient management, including all-oral antibiotic treatment, can be used for nonoperative treatment, including in children.

Our findings cannot be generalized to all patients with appendicitis. CODA trial enrollment criteria needed to be met, and ED discharge was predicated on patient stability and reliability for follow-up. In addition, these results only apply to those who can be discharged within 24 hours. Five percent of participants randomized to the antibiotic group progressed or changed their treatment preference and underwent appendectomy before 24 hours of observation. The potential for early worsening highlights the importance of serial evaluation before ED discharge is considered.

### Limitations

This study has limitations. First, although our adjusted analysis did not find outpatient management to be associated with an increased appendectomy risk, unaccounted factors may have differentiated patients discharged early from the hospitalized group. A similar adjusted analysis of SAEs could not be performed because of their rarity. These findings, however, support the safety of clinician selection across a broad range of patients. Second, this was not a randomized trial in which participants who qualified for outpatient care were assigned to early discharge or inpatient care, so we did not directly assess comparative effectiveness. Third, we recognize that there may be different perspectives on the time threshold to define outpatient management and hospitalization. Our definitions, discharge within or after 24 hours of presentation, are commonly used and are based on when participants received at least their first antibiotic dose and on CMS criteria.^7,8,9^ Thus, these definitions can be generalized and have cost implications in the US. We also found similar results for participants discharged within 12 hours.

## Conclusions

The findings of this cohort study support that outpatient antibiotic management is safe for selected adults with acute appendicitis. It appears that most patients who choose antibiotics can avoid hospitalization without incurring increased risk of serious complications or appendectomy. Outpatient management should be included in shared decision-making discussions of patient preferences for outcomes associated with nonoperative and operative care.

## Notes

Supplement 1. **eAppendix.** Variables Used in Multiple Imputations **eTable.** Characteristics of CODA Trial Participants Randomized to Antibiotics with Appendectomy Within 24 Hours of Emergency Department Arrival **eFigure 1.** Randomization and Disposition of Participants in the CODA Trial **eFigure 2.** Cumulative Incidence of Appendectomy Through Two Years in the Antibiotic Group by Emergency Department Discharge Within 24 Hours or Later

Supplement 2.
Nonauthor Collaborators
