# Supplementary material for: Analysis of Outcomes Associated With Outpatient Management of Nonoperatively Treated Patients With Appendicitis
Source: JAMA Netw Open. 2022 Jul 1;5(7):e2220039. doi: 10.1001/jamanetworkopen.2022.20039 (PMC9250049; doi:10.1001/jamanetworkopen.2022.20039)
Supplement: Supplement 1. — eAppendix. Variables Used in Multiple Imputations eTable. Characteristics of CODA Trial Participants Randomized to Antibiotics with Appendectomy Within 24 Hours of Emergency Department Arrival eFigure 1. Randomization and Disposition of Participants in the CODA Trial eFigure 2. Cumulative Incidence of Appendectomy Through Two Years in the Antibiotic Group by Emergency Department Discharge Within 24 Hours or Later [file jamanetwopen-e2220039-s001.pdf]

Supplementary Online Content

Writing Group for the CODA Collaborative. Analysis of outcomes associated with outpatient management of nonoperatively treated patients with appendicitis. *JAMA Netw Open*. 2022;5(7):e2220039. doi:10.1001/jamanetworkopen.2022.20039

eAppendix. Variables Used in Multiple Imputations

eTable. Characteristics of CODA Trial Participants Randomized to Antibiotics with Appendectomy Within 24 Hours of Emergency Department Arrival

eFigure 1. Randomization and Disposition of Participants in the CODA Trial

eFigure 2. Cumulative Incidence of Appendectomy Through Two Years in the Antibiotic Group by Emergency Department Discharge Within 24 Hours or Later

This supplementary material has been provided by the authors to give readers additional information about their work.

© 2022 Talan DA et al. *JAMA Network Open.*

eAppendix. Variables Used in Multiple Imputations

Variables used in multiple imputations (all variables were obtained at index except where noted):

Early discharge status, appendectomy within 7 days, site, appendicolith on imaging, periappendiceal fat on imaging, periappendiceal fluid on imaging, appendiceal diameter on imaging, height (cm), weight (kg), diabetes, Alvarado score, Charlson score, EQ-5D, lives alone vs. with others, primary language (English/other or Spanish), insurance (commercial, Medicare/Tricare, Medicaid/state, or other/none), employment (employed, student, or unemployed/other), education (HS/GED or less, or some beyond HS/GED), number of adults in the household with income, number of dependents, sharing responsibilities of dependents, physical at work (most or all of the time, some of the time or less, or not employed), smoking history (yes or no), combined household income, Global Health questions 1-9 (each with a five-point scale rating, covering general health, quality of life, physical health, mental health, social activities/relationships, and everyday physical activities), health literacy help (yes or no), below poverty or Medicaid/state, age, sex, fever, nausea or vomiting, white blood cell count, maximum pain score recorded in the emergency department, and symptom duration.

© 2022 Talan DA et al. *JAMA Network Open.*

eTable. Characteristics of CODA Trial Participants Randomized to Antibiotics with Appendectomy Within 24 Hours of Emergency Department Arrival

	Appendectomy 0-24 hours (n=42)
	**n (%)**
**Age in years**	
<30	13 (31.0)
30-39	10 (23.8)
40-49	10 (23.8)
50-59	6 (14.3)
60-69	1 (2.4)
70-79	2 (4.8)
≥80	0 (0.0)
**Sex**	
Male	24 (57.1)
Female	18 (42.9)
**Race**[a]	
White	19 (46.3)
Black, African American	6 (14.6)
American Indian or Alaska Native	1 (2.4)
Asian	3 (7.1)
Native Hawaiian or Other Pacific Islander	0 (0.0)
Other race	10 (24.3)
Multiple races	2 (4.9)
**Hispanic**	
No	23 (54.8)
Yes	19 (45.2)
**Primary language**	
English	30 (71.4)
Spanish	10 (23.8)
Other	2 (4.8)
**Health insurance**	
Commercial	14 (33.3)
Medicare or Tricare	3 (7.1)
Medicaid or state	10 (23.8)
Other or none	14 (33.3)
**Health literacy help**[a]	
Never/rarely	32 (78.0)
Sometimes or more	9 (22.0)
**Below federal poverty level/Medicaid**[a]	
No	20 (69.0)

© 2022 Talan DA et al. *JAMA Network Open.*

Yes	9 (31.0)
**Dependents**	
No	15 (35.7)
Yes	27 (64.2)
**Body mass index, kg/m^2**[a]	
<25	7 (17.5)
25-35	28 (70.0)
>35	5 (12.5)
**Symptom duration, days**	
<1	7 (16.7)
1-<2	20 (47.6)
≥2	15 (35.7)
**Fever**	
No	31 (73.8)
Yes	11 (26.1)
**Nausea, vomiting, or anorexia**	
No	11 (26.2)
Yes	31 (73.8)
**CT only**	
No	6 (14.2)
Yes	36 (85.7)
**Ultrasound only**	
No	41 (97.6)
Yes	1 (2.4)
**Appendicolith**	
No	25 (59.5)
Yes	17 (40.5)
**Perforation, abscess, or phlegmon**[a]	
No or not mentioned	35 (85.3)
Yes	6 (14.6)
	**mean (SD)**
**Instrumental support**[b]	16 (4.7)
**Charlson score**[a]	0.4 (0.9)
**Alvarado score**[a]	7.0 (1.5)
**Pain score in past 7 days**	6.5 (3.1)
**White blood count, K/uL**[a]	14 (4.1)
**Appendiceal diameter, mm**[a]	12 (3.9)

CODA, Comparison of Antibiotic Drugs and Appendectomy; CT, computed tomography

© 2022 Talan DA et al. *JAMA Network Open.*

[a] Participants with missing data were excluded from the denominator. Missing data occurred for participants as follows: race - 1; health literacy help - 1; below federal poverty level - 13; body mass index - 2; perforation, abscess, or phlegmon - 1; instrumental support - 1; Charlson score - 0; Alvarado score -2; pain score - 0; white blood cell count - 0; and appendiceal diameter - 4.

[b] Instrumental support score was determined by PROMIS patient-related outcome of having someone to take the patient to the doctor, run errands, and help with daily chores and if confined to bed.[11]

© 2022 Talan DA et al. *JAMA Network Open.*

eFigure 1. Randomization and Disposition of Participants in the CODA Trial

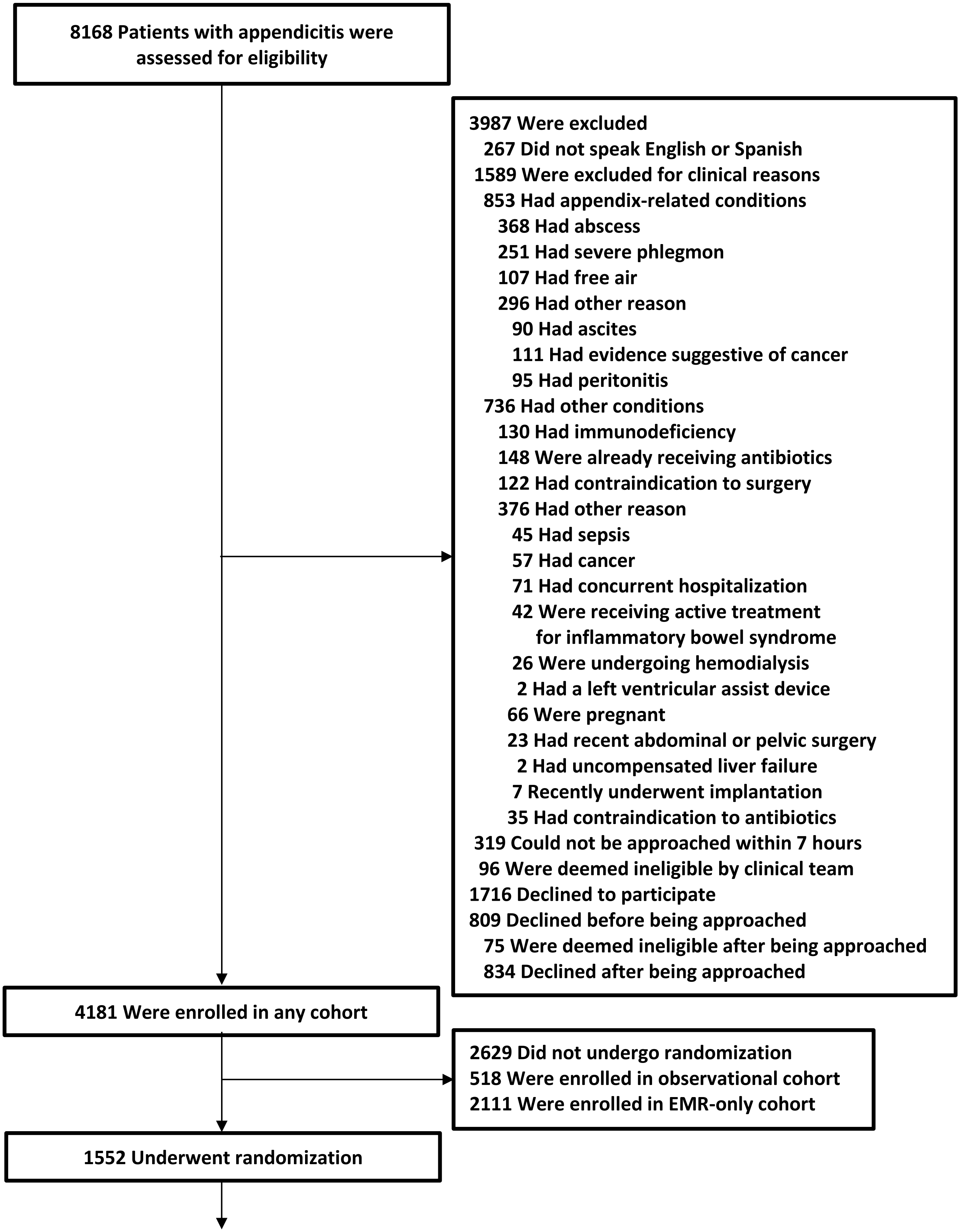

© 2022 Talan DA et al. *JAMA Network Open.*

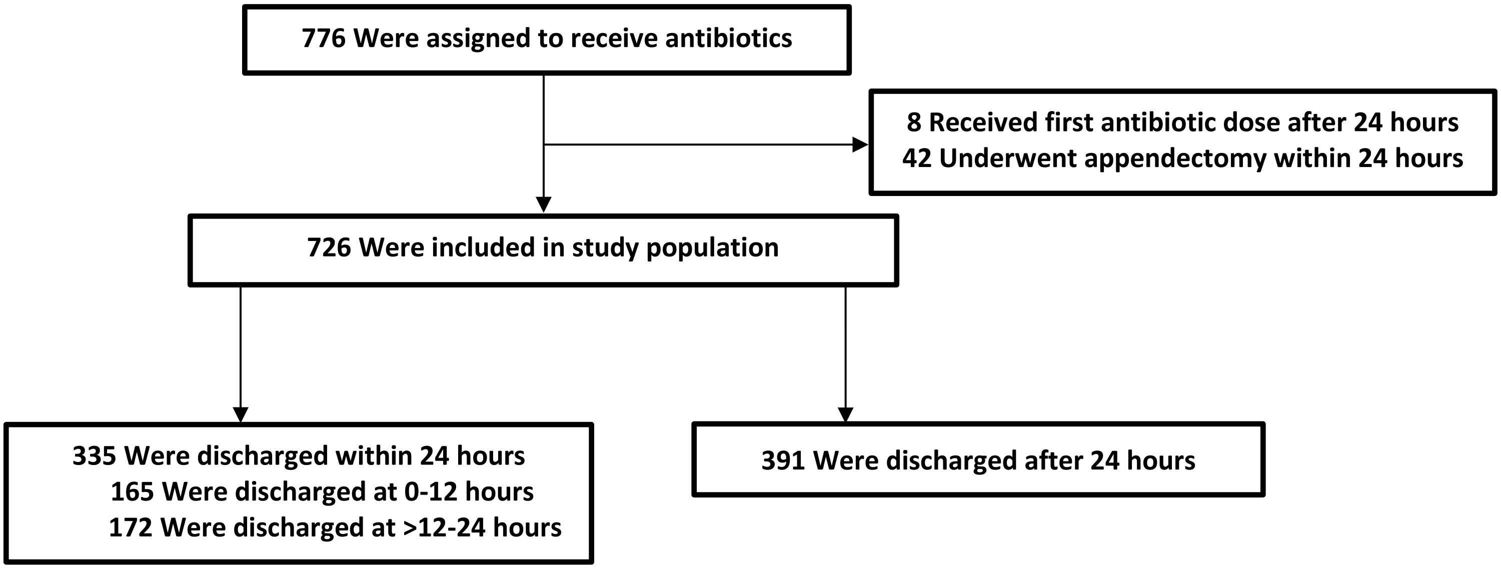

© 2022 Talan DA et al. *JAMA Network Open.*

eFigure 2. Cumulative Incidence of Appendectomy Through Two Years in the Antibiotic Group by Emergency Department Discharge within 24 Hours or Later

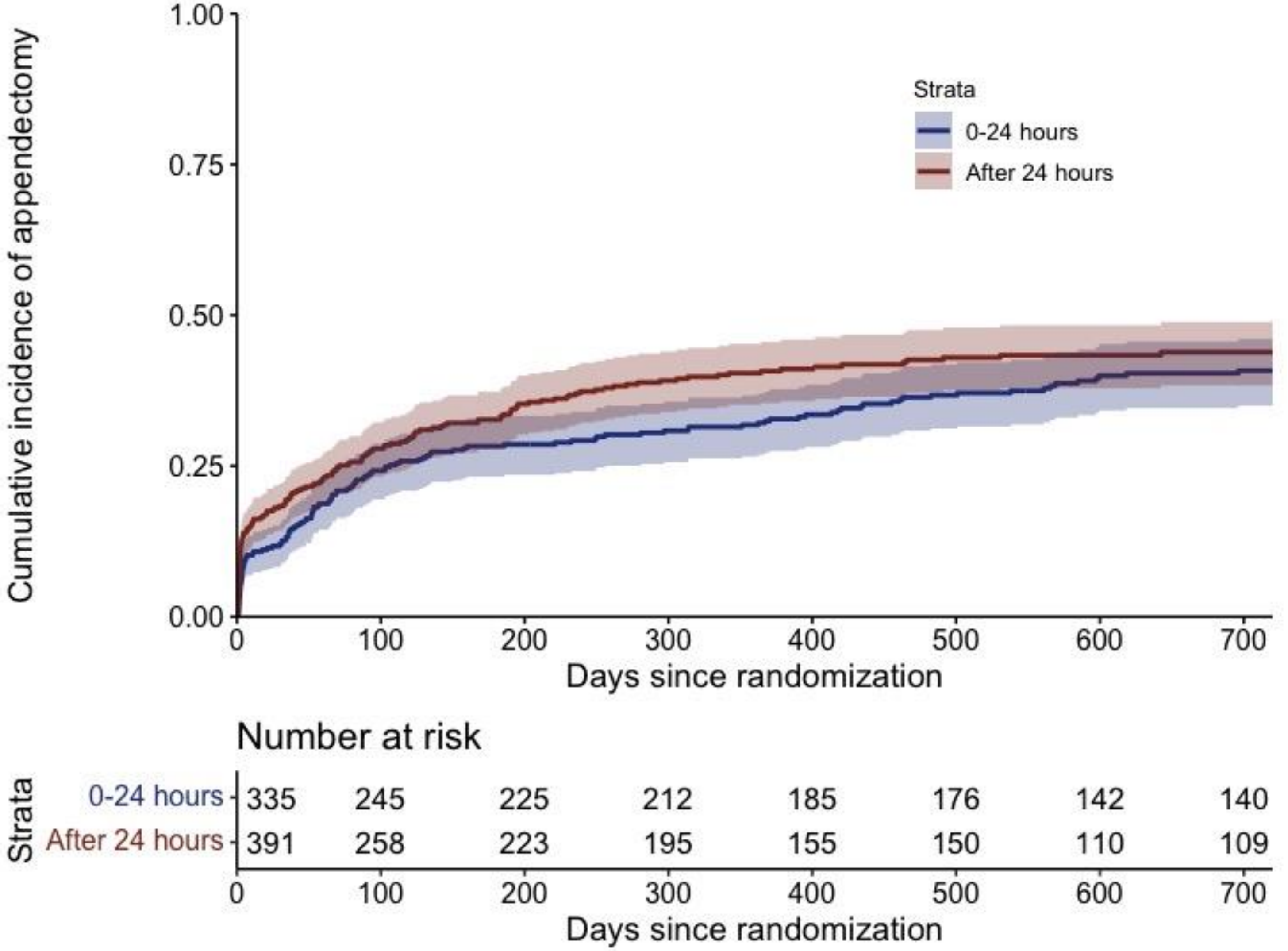

© 2022 Talan DA et al. *JAMA Network Open.*
