# Supplementary material for: Analysis of Outcomes Associated With Outpatient Management of Nonoperatively Treated Patients With Appendicitis
Source: JAMA Netw Open. 2022 Jul 1;5(7):e2220039. doi: 10.1001/jamanetworkopen.2022.20039 (PMC9250049; doi:10.1001/jamanetworkopen.2022.20039)
Supplement: Supplement 2. — Nonauthor Collaborators [file jamanetwopen-e2220039-s002.pdf]

Supplemental Online Content: Nonauthor Collaborators

*Indicates required information. Only first name, last name, and suffix will appear in PubMed.

*Group Name(s): Writing Group for the CODA Collaborative							
***First Name and Middle Initial(s)**	***Last Name**	***Suffix (eg, Jr, III)**	Academic Degrees	Institution	Location (city, state/province, country)	Role or Contribution, eg, chair, principal investigator	Group (if more than 1 Group listed in the byline) and/or Subgroup (eg, Steering Committee)
Charles	Parsons		MD	Beth Israel Deaconess Medical Center	Boston, MA	Co-Investigator	Clinical Coordinating Center
Stephen	Odom		MD	Beth Israel Deaconess Medical Center	Boston, MA	Co-Investigator	Clinical Coordinating Center
Randall	Cooper			Columbia University Medical Center	New York, NY	Co-Investigator	Clinical Coordinating Center
Katherine	Fischkoff		MD	Columbia University Medical Center	New York, NY	Co-Investigator	Clinical Coordinating Center
Brant	Putnam		MD	Harbor- University of California Los Angeles Medical Center	Los Angeles, CA	Co-Investigator	Clinical Coordinating Center
Dennis	Kim		MD	Harbor- University of California Los Angeles Medical Center	Los Angeles, CA	Co-Investigator	Clinical Coordinating Center
Erin C.	Howell		MD	Harbor- University of California Los Angeles Medical Center	Los Angeles, CA	Co-Investigator	Clinical Coordinating Center
Lara H.	Spence		MD	Harbor- University of California Los Angeles Medical Center	Los Angeles, CA	Co-Investigator	Clinical Coordinating Center
Ross	Fleischman		MD	Harbor- University of California Los Angeles Medical Center	Los Angeles, CA	Co-Investigator	Clinical Coordinating Center
Erika	Wolff		PhD	Harborview Medical Center	Seattle, WA	Co-Investigator	Executive Committee
Farhood	Farjah		MD, MPH	Harborview Medical Center	Seattle, WA	Co-Investigator	
Hikmatullah	Arif			Harborview Medical Center	Seattle, WA	Study Staff	Clinical Coordinating Center
Kelsey	Pullar		MPH	Harborview Medical Center	Seattle, WA	Study Staff	Clincial Coordinating Center
Laura	Hennessey		RN	Harborview Medical Center	Seattle, WA	Study Staff	Clinical Coordinating Center
Alyssa	Hayward			Henry Ford Health Hospital	Detroit, MI	Study Staff	Clinical Coordinating Center
Lillian Adrianna	Hayes			Henry Ford Health Hospital	Detroit, MI	Study Staff	Clinical Coordinating Center
Vance	Sohn		MD	Madigan Army Medical Center	Joint Base Lewis-McChord,	Co-Investigator	Clinical Coordinating Center
Bruce	Chung		MD	Maine Medical Center	Portland, ME	Co-Investigator	Clinical Coordinating Center
Damien	Carter		MD	Maine Medical Center	Portland, ME	Co-Investigator	Clinical Coordinating Center
David	MacKenzie		MD	Maine Medical Center	Portland, ME	Co-Investigator	Clinical Coordinating Center
Debra	Burris		RN	Maine Medical Center	Portland, ME	Study Staff	Clinical Coordinating Center
Joseph	Mack		MD	Maine Medical Center	Portland, ME	Co-Investigator	Clinical Coordinating Center
Terilee	Gerry		MS, RN	Maine Medical Center	Portland, ME	Study Staff	Clinical Coordinating Center
Darin	Saltzman		MD, PhD	Olive View- University of California Los Angeles Medical Center	Los Angeles, CA	Co-Investigator	Clinical Coordinating Center
Debbie	Mireles		NP	Olive View- University of California Los Angeles Medical Center	Los Angeles, CA	Study Staff	Clinical Coordinating Center
Formosa	Chen		MD, MPH	Olive View- University of California Los Angeles Medical Center	Los Angeles, CA	Co-Investigator	Clinical Coordinating Center
Kavitha	Pathmarajah		MPH	Olive View- University of California Los Angeles Medical Center	Los Angeles, CA	Study Staff	Clinical Coordinating Center

Supplemental Online Content: Nonauthor Collaborators

*Indicates required information. Only first name, last name, and suffix will appear in PubMed.

***First Name and Middle Initial(s)**	***Last Name**	***Suffix (eg, Jr, III)**	Academic Degrees	Institution	Location (city, state/province, country)	Role or Contribution, eg, chair, principal investigator	Group (if more than 1 Group listed in the byline) and/or Subgroup (eg, Steering Committee)
Paul J	Schmit		MD	Olive View- University of California Los Angeles Medical Center	Los Angeles, CA	Co-Investigator	Clinical Coordinating Center
Robert	Bennion		MD	Olive View- University of California Los Angeles Medical Center	Los Angeles, CA	Co-Investigator	Clinical Coordinating Center
Melinda	Gibbons		MD	Olive View- University of California Los Angeles Medical Center	Los Angeles, CA	Co-Investigator	Clinical Coordinating Center
Bruce	Wolfe		MD	Oregon Health & Science University	Portland, OR	Clinical Reviewer	Data Safety and Monitoring Board
Elliott	Skopin		BS	PAB	Seattle, WA	Advisor	Patient Advisory Board
Heather	VanDusen		BS	PAB	Seattle, WA	Advisor	Patient Advisory Board
Kimberly	Deeney		BA	PAB	Medford, MA	Advisor	Patient Advisory Board
Mary	Guiden		BA	PAB	Fort Collins, CO	Advisor	Patient Advisory Board
Meridith	Weiss		MPH	PAB	Seattle, WA	Advisor	Patient Advisory Board
Miriam	Hernandez			PAB		Advisor	Patient Advisory Board
Brandon	Tudor		MD	Providence Regional Medical Center Everett	Everett, Washington	Co-Investigator	Clinical Coordinating Center
Careen	Foster		MD	Providence Regional Medical Center Everett	Everett, Washington	Co-Investigator	Clinical Coordinating Center
Shaina	Schaetzel		MD	Providence Regional Medical Center Everett	Everett, Washington	Co-Investigator	Clinical Coordinating Center
Arden	Morris		MD, MPH	Standford University	Stanford, CA	Clinical Reviewer	Data Safety and Monitoring Board
Dayna	Morgan		MSNBC	Swedish Medical Center	Seattle, WA	Study Staff	Clinical Coordinating Center
John	Tschirhart		MD	Swedish Medical Center	Seattle, WA	Co-Investigator	Clinical Coordinating Center
Julie	Wallick		BS, BA	Swedish Medical Center	Seattle, WA	Study Staff	Clinical Coordinating Center
Katherine	Mandell		MD, MPH	Swedish Medical Center	Seattle, WA	Co-Investigator	Clinical Coordinating Center
Ryan	Martinez		MD	Swedish Medical Center	Seattle, WA	Co-Investigator	Clinical Coordinating Center
Sean	Wells		MD	Swedish Medical Center	Seattle, WA	Co-Investigator	Clinical Coordinating Center
Steven	Steinberg		MD	The Ohio State University Wexner Medical Center	Columbus, OH	Co-Investigator	Clinical Coordinating Center
Jason	Maggi		MD	Tisch Hospital NYU Langone Medical Center	New York, NY	Co-Investigator	Clinical Coordinating Center
Kristyn	Pierce		MS	Tisch Hospital NYU Langone Medical Center	New York, NY	Co-Investigator	Clinical Coordinating Center
Marcovalerio	Melis		MD	Tisch Hospital NYU Langone Medical Center	New York, NY	Co-Investigator	Clinical Coordinating Center
Mohamad	Abouzeid		MD	Tisch Hospital NYU Langone Medical Center	New York, NY	Co-Investigator	Clinical Coordinating Center
Paresh	Shah		MD	Tisch Hospital NYU Langone Medical Center	New York, NY	Co-Investigator	Clinical Coordinating Center

*Indicates required information. Only first name, last name, and suffix will appear in PubMed.

*First Name and Middle Initial(s)	*Last Name	*Suffix (eg, Jr, III)	Academic Degrees	Institution	Location (city, state/province, country)	Role or Contribution, eg, chair, principal investigator	Group (if more than 1 Group listed in the byline) and/or Subgroup (eg, Steering Committee)
Prashant	Sinha		MD	Tisch Hospital NYU Langone Medical Center	New York, NY	Co-Investigator	Clinical Coordinating Center
Cathy	Fairfield		BSN	University of Iowa Healthcare	Iowa City, IA	Study Staff	Clincial Coordinating Center
Dionne	Skeete		MD	University of Iowa Healthcare	Iowa City, IA	Co-Investigator	Clincial Coordinating Center
Cindy	Hsu		MD, PHD	University of Michigan Medical Center	Ann Arbor, MI	Co-Investigator	Clinical Coordinating Center
Krishnan	Raghavendran		MBBS, MS	University of Michigan Medical Center	Ann Arbor, MI	Study Staff	Clinical Coordinating Center
Nathan	Haas		MD	University of Michigan Medical Center	Ann Arbor, MI	Co-Investigator	Clinical Coordinating Center
Norman	Olbrich			University of Michigan Medical Center	Ann Arbor, MI	Study Staff	Clinical Coordinating Center
Pauline	Park		MD	University of Michigan Medical Center	Ann Arbor, MI	Co-Investigator	Clinical Coordinating Center
Hasan	Alam		MD	University of Michigan Medical Center	Ann Arbor, MI	Co-Investigator	Clinical Coordinating Center
Deepti	Patki		MS	University of Mississippi Medical Center	Jackson, MS	Study Staff	Clinical Coordinating Center
Rebekah K.	Peacock		RN	University of Mississippi Medical Center	Jackson, MS	Study Staff	Clinical Coordinating Center
Donald M.	Yealy		MD	University of Pittsburgh Medical Center	Pittsburgh, PA	Clinical Reviewer	Data Safety and Monitoring Board
Debbie	Lew			University of Texas Lyndon B. Johnson General Hospital	Houston, TX	Study Staff	Clinical Coordinating Center
Karla	Bernardi		MD	University of Texas Lyndon B. Johnson General Hospital	Houston, TX	Co-Investigator	Clinical Coordinating Center
Naila	Dhanani		MD	University of Texas Lyndon B. Johnson General Hospital	Houston, TX	Co-Investigator	Clinical Coordinating Center
Oscar	Olavarria		MD	University of Texas Lyndon B. Johnson General Hospital	Houston, TX	Co-Investigator	Clinical Coordinating Center
Stephanie	Marquez			University of Texas Lyndon B. Johnson General Hospital	Houston, TX	Co-Investigator	Clinical Coordinating Center
Tien C.	Ko		MD	University of Texas Lyndon B. Johnson General Hospital	Houston, TX	Co-Investigator	Clinical Coordinating Center
Amber	Sabbatini		MD, MPH	University of Washington	Seattle, WA	Co-Investigator	Clinical Coordinating Center
Estell	Williams		MD	University of Washington	Seattle, WA	Co-Investigator	Clinical Coordinating Center
Karen	Horvath		MD	University of Washington	Seattle, WA	Co-Investigator	Clinical Coordinating Center
Zoe	Parr		MD	University of Washington	Seattle, WA	Co-Investigator	Clinical Coordinating Center
Karen F.	Miller		RN, MPA	Vanderbilt University Medical Center	Nashville, Tennessee	Study Staff	Clinical Coordinating Center

Supplemental Online Content: Nonauthor Collaborators

*Indicates required information. Only first name, last name, and suffix will appear in PubMed.

***First Name and Middle Initial(s)**	***Last Name**	***Suffix (eg, Jr, III)**	Academic Degrees	Institution	Location (city, state/province, country)	Role or Contribution, eg, chair, principal investigator	Group (if more than 1 Group listed in the byline) and/or Subgroup (eg, Steering Committee)
Kelly M.	Moser			Vanderbilt University Medical Center	Nashville, Tennessee	Study Staff	Clinical Coordinating Center
Abigail	Wiebusch		MD	Virginia Mason Medical Center	Seattle, WA	Co-Investigator	Clinical Coordinating Center
Julianna	Yu		MD	Virginia Mason Medical Center	Seattle, WA	Co-Investigator	Clinical Coordinating Center
Scott	Osborn		MD	Virginia Mason Medical Center	Seattle, WA	Co-Investigator	Clinical Coordinating Center
Billie	Johnsson		MS	Weill Cornell Medical Center	New York, NY	Co-Investigator	Clinical Coordinating Center
Karla	Ballman		PhD	Weill Cornell Medical Center	New York, NY	Clinical Reviewer	Data Safety and Monitoring Board
Lauren	Mount		MD	Weill Cornell Medical Center	New York, NY	Co-Investigator	Clinical Coordinating Center
Robert J.	Winchell		MD	Weill Cornell Medical Center	New York, NY	Co-Investigator	Clinical Coordinating Center
Sunday	Clark		ScD, MPH	Weill Cornell Medical Center	New York, NY	Co-Investigator	Clinical Coordinating Center
Thomas	Diflo		MD	Westchester Medical Center	Valhalla, NY	Clinical Reviewer	Data Safety and Monitoring Board
Kathleen	O'Connor		EdD			Clinical Reviewer	Data Safety and Monitoring Board
Olga	Owens		N-PC		Las Vegas, NV	Clinical Reviewer	Data Safety and Monitoring Board
